# Dynamic Supraspliceosomes Are Assembled on Different Transcripts Regardless of Their Intron Number and Splicing State

**DOI:** 10.3389/fgene.2020.00409

**Published:** 2020-05-15

**Authors:** Naama Sebbag-Sznajder, Yehuda Brody, Hodaya Hochberg-Laufer, Yaron Shav-Tal, Joseph Sperling, Ruth Sperling

**Affiliations:** ^1^Department of Genetics, The Hebrew University of Jerusalem, Jerusalem, Israel; ^2^The Mina and Everard Goodman Faculty of Life Sciences and The Institute of Nanotechnology and Advanced Materials, Bar Ilan University, Ramat Gan, Israel; ^3^Department of Organic Chemistry, The Weizmann Institute of Science, Rehovot, Israel

**Keywords:** pre-mRNA splicing, specific supraspliceosomes, MS2-tagged supraspliceosomes, splicing inhibition, splicing factors

## Abstract

Splicing and alternative splicing of pre-mRNA are key sources in the formation of diversity in the human proteome. These processes have a central role in the regulation of the gene expression pathway. Yet, how spliceosomes are assembled on a multi-intronic pre-mRNA is at present not well understood. To study the spliceosomes assembled *in vivo* on transcripts with variable number of introns, we examined a series of three related transcripts derived from the β-globin gene, where two transcript types contained increasing number of introns, while one had only an exon. Each transcript had multiple MS2 sequence repeats that can be bound by the MS2 coat protein. Using our protocol for isolation of endogenous spliceosomes under native conditions from cell nuclei, we show that all three transcripts are found in supraspliceosomes – 21 MDa dynamic complexes, sedimenting at 200S in glycerol gradients, and composed of four native spliceosomes connected by the transcript. Affinity purification of complexes assembled on the transcript with most introns (termed E6), using the MS2 tag, confirmed the assembly of E6 in supraspliceosomes with components such as Sm proteins and PSF. Furthermore, splicing inhibition by spliceostatin A did not inhibit the assembly of supraspliceosomes on the E6 transcript, yet increased the percentage of E6 pre-mRNA supraspliceosomes. These findings were corroborated in intact cells, using RNA FISH to detect the MS2-tagged E6 mRNA, together with GFP-tagged splicing factors, showing the assembly of splicing factors SRSF2, U1-70K, and PRP8 onto the E6 transcripts under normal conditions and also when splicing was inhibited. This study shows that different transcripts with different number of introns, or lacking an intron, are assembled in supraspliceosomes even when splicing is inhibited. This assembly starts at the site of transcription and can continue during the life of the transcript in the nucleoplasm. This study further confirms the dynamic and universal nature of supraspliceosomes that package RNA polymerase II transcribed pre-mRNAs into complexes composed of four native spliceosomes connected by the transcript, independent of their length, number of introns, or splicing state.

## Introduction

To generate an mRNA, RNA polymerase II (Pol II) transcribed pre-mRNAs must go through nuclear processing events prior to their export into the cytoplasm. RNA processing is composed of 5′-end capping, 3′-end processing, splicing, and RNA editing. Pre-mRNA splicing takes place in a dynamic ribonucleoprotein complex (RNP) – the spliceosome. The splicing machinery engages with *cis* elements in the pre-mRNA such as the 5′ and 3′ splice sites (SSs) consensus sequences, a branch site, a polypyrimidine tract, and exonic and intronic splicing enhancers and silencers (reviewed in Wahl et al., 2009; Will and Luhrmann, 2011; Papasaikas and Valcarcel, 2016). The *cis* elements in the pre-mRNA are identified by *trans* factors, such as the U1, U2, U4, U5, and U6 snRNPs, and many splicing factors, including the hnRNP proteins and the serine/arginine (SR)-rich protein family.

The splicing reaction is a two-step transesterification process that is performed by the spliceosome. Spliceosome assembly can be monitored *in vitro*, showing that this is a process occurring in a stepwise manner, generating intermediate complexes (reviewed in Brow, 2002; Will and Luhrmann, 2011). The spliceosomal U snRNPs, which are key players in pre-mRNA splicing, go through major dynamic alterations in their RNA:RNA contacts during the assembly of the spliceosome and the splicing reaction. The first step is the base pairing of U1 snRNP with the 5′ splice site and is followed by the assembly of additional snRNPs. The U snRNPs also interact with numerous splicing factors during the assembly of the spliceosome and the splicing reaction. The interaction of snRNPs with the pre-mRNA is supported by proteins from the SR protein family. These are SR-rich proteins (Valcarcel and Green, 1996; Shepard and Hertel, 2009), and they are required for the stabilization of the early spliceosomal complex. For instance, the interaction of SRSF2 with U1 snRNP is assisted by the U1 snRNP protein U1-70K (Wu and Maniatis, 1993; Zahler and Roth, 1995) and can determine transcript fate (Fu et al., 1992). Recent subnanometric structures of splicing complexes determined by high-resolution cryo-EM have portrayed the catalytic center of the spliceosome and have revealed the dynamic alterations in U snRNA:U snRNA and U snRNA:pre-mRNA interactions taking place during the assembly of the spliceosomes and the splicing reaction, which is reflected in alterations in the structures of spliceosome intermediates. A key protein, present at the heart of the spliceosome, is the U5 snRNP protein PRP8 (reviewed in Shi, 2017a,b; Fica et al., 2017; Wilkinson et al., 2018; Plaschka et al., 2019; Yan et al., 2019).

The majority of Poll II transcribed pre-mRNAs have multiple introns, and they can thus undergo alternative splicing (AS), which is a key element in the regulation of gene expression (reviewed in Kelemen et al., 2013; Akerman et al., 2015; Lee and Rio, 2015; Naftelberg et al., 2015). Furthermore, errors in AS are at the heart of numerous human diseases, as well as in cancer (Irimia and Blencowe, 2012; Singh and Cooper, 2012; Chabot and Shkreta, 2016). Splicing regulation requires multiple interactions between sequences present in the pre-mRNA and *trans* factors that target these positive and negative signals. Among the *trans* factors are the SR proteins (Lin and Fu, 2007; Long and Caceres, 2009; Shepard and Hertel, 2009; Han et al., 2011) and the hnRNP proteins (Han et al., 2010; Busch and Hertel, 2012). The accuracy of splice site selection is accomplished through the blending of numerous weak interactions between RNA:RNA, protein:RNA, and protein:protein.

The endogenous spliceosome assembles individual transcripts of Pol II in a giant RNP (21 MDa)—called the supraspliceosome. All nuclear pre-mRNAs, regardless of their intron number and length, are packaged in supraspliceosomes. The latter can be isolated from cell nuclei under physiological conditions and remain active in splicing (reviewed in Sperling et al., 2008; Shefer et al., 2014; Sperling, 2017). Supraspliceosomes are composed of the five spliceosomal U snRNPs and additional splicing factors (Miriami et al., 1995; Azubel et al., 2006). The five spliceosomal U snRNPs are associated with the supraspliceosome at all splicing steps, as revealed by examining affinity-purified specific supraspliceosomes at different splicing stages (Kotzer-Nevo et al., 2014). The supraspliceosome harbors splicing factors such as all phosphorylated SR proteins (Yitzhaki et al., 1996), hnRNP G (Heinrich et al., 2009), and the alternative splicing factors RBM4 and WT1 (Markus et al., 2006) and ZRANB2 (Yang et al., 2013). Mass spectrometry (MS) analysis of supraspliceosomes has revealed further splicing factor components (Chen et al., 2007) as did MS analysis of specific supraspliceosomes analyzed at distinct functional states (Kotzer-Nevo et al., 2014). The presence of regulatory splicing factors in supraspliceosomes is in accordance with their task in splicing and AS (Heinrich et al., 2009; Sebbag-Sznajder et al., 2012). Additional components found in supraspliceosomes are pre-mRNA processing factors, among them are the cap-binding proteins, 3′-end processing components (Raitskin et al., 2002), and the ADAR1 and ADAR2 editing enzymes (Raitskin et al., 2001). These findings portray the supraspliceosome as the nuclear pre-mRNA processing machine.

The supraspliceosome is formed of four active native spliceosomes joined together by the pre-mRNA (Sperling et al., 1997; Müller et al., 1998; Medalia et al., 2002; Azubel et al., 2004; Azubel et al., 2006; Cohen-Krausz et al., 2007). The native spliceosome, which is similar to an *in vitro* assembled spliceosome, is an elongated globular particle made of large and small substructures, as resolved by single particle cryo-electron microscopy (cryo-EM) at a resolution of 20 Å (Azubel et al., 2004). *In silico* studies have localized the spliceosomal U snRNPs within the native spliceosome in a single layout, mainly within the large substructure, thereby protecting the elements of the active center in the cleft within the spliceosome (Frankenstein et al., 2012). The native spliceosomes are placed within the supraspliceosome with their small substructures facing its center, an arrangement that enables interactions between them. Communication between the native spliceosomes within the supraspliceosomes is likely an essential aspect of splicing control, also required for quality control of the mRNAs (Azubel et al., 2006; Cohen-Krausz et al., 2007). The supraspliceosome thus emerges as a principal controller of pre-mRNA processing important in the regulation of multiple pre-mRNA processing steps.

Although most RNA Pol II transcribed transcripts are multi-intronic, at present, it is not well understood how spliceosomes are assembled on a multi-intronic pre-mRNA. One view that has emerged from *in vitro* studies has suggested that a spliceosome is assembled onto each of the synthesized introns of the pre-mRNA, which disassembles after splicing is performed in order to get ready for the next round of splicing (Staley and Guthrie, 1998). This model suggests that splicing of a multi-intronic pre-mRNA requires the assembly of multiple spliceosomes, whose number equals the number of introns. Another view, developed from studies of the endogenous spliceosome, demonstrated that the pre-mRNA is assembled into supraspliceosomes, composed of four active native spliceosomes, which are linked by the transcript. Notably, transcripts having diverse number of introns or length are found in supraspliceosomes, indicating that the four spliceosomes of the supraspliceosome are adequate for splicing of every transcript. Furthermore, the distinctive size and hydrodynamic assets of supraspliceosomes signify their universal nature (reviewed in Sperling et al., 2008; Shefer et al., 2014; Sperling, 2017; Sperling and Sperling, 2017). To study the spliceosomes assembled *in vivo* on transcripts with variable number of introns, we examined herein a series of three related transcripts: two with rising number of introns originated from the β-globin gene, while one had only an exon. Each transcript had multiple MS2 sequence repeats that can be bound by the MS2 coat protein (Brody et al., 2011). We show here that pre-mRNA transcripts with no intron (termed E1), with two introns (E3), and with five introns (E6) are found in supraspliceosomes. Affinity purification of complexes assembled on the transcript with most introns (termed E6), using the MS2 tag, confirmed the assembly of E6 mRNA in supraspliceosomes. Furthermore, splicing inhibition by spliceostatin A did not inhibit the assembly of supraspliceosomes on the E6 transcript, yet, increased the percentage of E6 pre-mRNA supraspliceosomes. These findings were corroborated in intact cells, using RNA FISH to detect the MS2-tagged E6 mRNA, together with GFP-tagged splicing factors, showing the assembly of splicing factors SRSF2, U1-70K, and PRP8 onto the E6 transcripts under normal conditions and also when splicing was inhibited. This study shows that different transcripts with different number of introns, or lacking one, are assembled in supraspliceosomes even when splicing is inhibited. This assembly starts at the site of transcription and continues during the life of the transcript in the nucleoplasm. This study further confirms the dynamic and universal nature of supraspliceosomes that package Pol II transcribed pre-mRNAs into complexes composed of four native spliceosomes connected by the transcript, independent of their length, number of introns, or splicing state.

## Materials and Methods

### Cells

U2OS Tet-On human osteosarcoma cells were grown in low-glucose Dulbecco’s modified Eagle’s medium (DMEM, Biological Industries, Israel) containing 10% fetal bovine serum (FBS, HyClone). A series of U2OS stable cell lines were used, as described in Brody et al. (2011). The cells used were U2OS Tet-On cells containing a stable integration of a Tet-inducible β-globin mini-gene termed E1, E3, or E6, where the number denotes the number of exons in the gene. The genes were integrated as tandem gene arrays in one locus that forms a detectable site of transcription upon gene activation. Induction of transcription was obtained by doxycycline (dox, 1 μg/mL, Sigma) and results in the expression of a transcript encoding β-globin fused to a CFP protein that contains an SKL tripeptide for peroxisomal targeting, and in the 3′UTR, a series of 18 × MS2 sequence repeats. For imaging, U2OS E6 cells carrying a stable integration of BACs were used. The BACs were C-terminally GFP-tagged SC35 (SRSF2), U1-70K, and PRP8 that were previously described (Poser et al., 2008; Huranova et al., 2010; Hochberg-Laufer et al., 2019b). For splicing inhibition, U2OS E6 cells were incubated with either 10 or 100 ng/mL Spliceostatin (SSA) (a kind gift from Dr. Yoshida’s lab) for 5 h, or with Pladienolide B (10 μM, Santa Cruz) for 6 h.

### Supraspliceosome Isolation

For isolation of supraspliceosomes, first, nuclear supernatants, which were enriched in supraspliceosomes were prepared as previously described (Miriami et al., 1995; Azubel et al., 2006), from the U2OS cell clones. Briefly, nuclear supernatants enriched for supraspliceosomes were prepared from purified nuclei of the above-described U2OS cells, by microsonication of the nuclei and precipitation of the chromatin in the presence of tRNA. After fractionation of the nuclear supernatants in 10–45% glycerol gradients in an SW41 rotor, at 11,700 rpm for 18 h, the gradients were analyzed by EM visualization of aliquots from fractions corresponding to the 200S region of the gradient (tobacco mosaic virus served as a sedimentation coefficient marker). RNA extraction from gradient fractions was performed as previously described (Azubel et al., 2006).

### RNA Isolation and Analysis

RNA was isolated from each gradient fraction (520 μL) by adding 150 μL of extraction buffer (50 mM Tris–HCl, pH 7.5, 30 mM NaCl, and 1 mM EDTA) and 50 μL of 10% SDS. RNA isolated from gradient fractions was analyzed by RT-PCR as previously described (Sebbag-Sznajder et al., 2012).

For total RNA analysis, RNA was extracted (Sperling et al., 1985) and RT-PCR was performed with the relevant primers corresponding to the E1, E3, and E6 mRNA, and actin as a control.

For analysis of E1:

CFP-SKL forward: 5′-GCAAGCTGACCCTGAAGTTC-3′
CFP-SKL reverse: 5′-GTCTTGTAGTTGCCGTCGT-3′

For analysis of E3 and E6:

E6 β-globin forward Ex1: 5′-GCAACCTCAAACAGACA CCA-3′
E6 β-globin reverse Ex2: 5′-CAGCATCAGGAGTGGAC AGA-3′
E6 β-globin reverse CFP: 5′-GCCCTTGCTCACCATGAAT-3′

For analysis of β-actin:

Actin sense: 5′-CAAGGCCAACCGCGAGAAGATGAC-3′
Actin antisense: 5′-AGGAAGGAAGGCTGGAAGAGTGC-3′

### Affinity Purification of MS2-Tagged E6 Supraspliceosomes

1.5 mL of supraspliceosomes isolated from U2OS E6 cells (from 10 plates of 15 cm, collecting fractions 8–10 of the glycerol gradients, see above) were incubated with 300 μg of MS2-MBP [prepared as described (Das et al., 2000)] for 1 h, at 4°C. Next, washed Amylose beads (200 μL of Amylose resin 50% in 20% EtOH) were added to the supraspliceosomes/MS2-MBP sample and left for overnight incubation at 4°C with shaking at 15 rpm. As a control, we used supraspliceosomes without MS2-MBP. After centrifugation of the beads with supraspliceosomes (supernatant termed unbound), and washing (×5), supraspliceosomes were eluted with maltose by incubating with 400 μL of the elution buffer (20 mM maltose) for 30 min at 15 rpm, at 4°C. The tubes were spun down and the supernatant was kept for analysis.

### Western Blotting

For WB analysis, proteins were precipitated in 80% cold acetone, using 1 μL of Quick Precip (EdgeBio, cat No 14201) as carrier. The pelleted proteins were dissolved in SDS sample buffer and analyzed by 12% SDS PAGE. Gels were either stained with Coomassie Blue G250, or analyzed by Western blots using the anti-Sm antibody [Y12, 1: 10,000 dilution in NET (150 mM NaCl, 50 mM Tris, pH 7.5, 0.05% Triton X-100/or NP-40)]; anti-S14 Mab (1:3000 dilution); and anti-PSF Mab (SFPQ) (Sigma, 1:3000 dilution), visualized with horseradish peroxidase conjugated to goat anti-mouse antibody (1:3000 dilution of goat anti-mouse Fab2-HRP, Jackson), as previously described (Sebbag-Sznajder et al., 2012).

### EM Visualization

Aliquots (10 μL) from the samples were absorbed on glow-discharged carbon-coated copper EM grids, washed with water, and negatively stained with 1% (w/v) uranyl-acetate. A Tecnai 12 TEM (FEI), operating at an acceleration voltage of 100 kV, equipped with a CCD camera was used.

### Fluorescence *in situ* Hybridization

Cells grown on coverslips were fixed for 20 min in 4% PFA and then transferred to 70% ethanol at 4°C for overnight. The next day, cells were washed with 1 × PBS and treated for 2.5 min with 0.5% Triton X-100. Cells were washed with 1 × PBS and incubated for 10 min in 40% formamide (4% SSC). Cells were transferred to 40% formamide at 37°C and hybridized overnight with a specific Cy5 fluorescently labeled DNA probe (∼10 ng probe, 50 mer) that binds to the MS2 region of the MS2 repeats. The intron probe was described in Brody et al. (2011). The next day, cells were washed twice with 40% formamide for 15 min and then washed for 2 h in 1 × PBS. Nuclei were counterstained with Hoechst 33342 (Sigma) and coverslips were mounted in mounting medium. Wide-field fluorescence images were obtained using the Cell^R system based on an Olympus IX81 fully motorized inverted microscope (60 × PlanApo objective, 1.42 NA) fitted with an Orca-AG CCD camera (Hamamatsu) driven by the Cell^R software. When imaging, the focus was on the active transcription sites that usually produced a strong signal in the nucleus. Presentation of this transcription site signal without saturation usually reduced the ability to present the weaker mRNA signal in the rest of the cell.

## Results

### A Series of Transcripts With Variable Number of Introns

**FIGURE 1:**
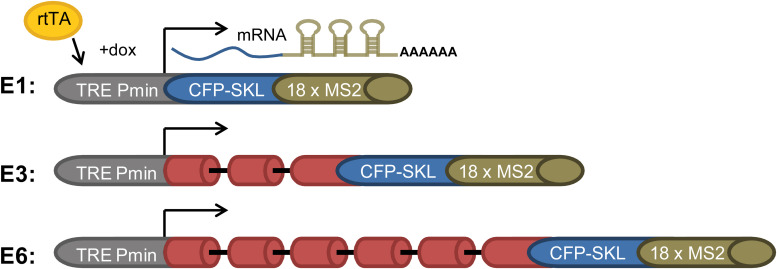
E1, E3, and E6 globin constructs. (i) E1—a gene with no introns containing part of exon 3 and the CFP-SKL signal. The 3′UTR has 18 MS2 repeats; (ii) E3—having two introns and three exons, and identical 3′-end to that of E1; and (iii) E6—in which part of exon 2 intron two and part of exon 3 were duplicated three times and fused to the 5′ part of exon 3, with the 3′-end identical to E1 and E3, generating a construct with six exons and five introns. All constructs were Tet-On inducible and stably integrated into human U2OS Tet-On cells (Brody et al., 2011). Transcription was induced by the addition of doxycycline (dox) to the medium in the presence of the rtTA (Tet-On) transactivator. The different-sized gene constructs produced the correctly spliced mRNAs.

Splicing and alternative splicing of pre-mRNA play a major role in regulating gene expression. Yet, how spliceosomes are assembled on a multi-intronic pre-mRNA is at present not well understood. To study the spliceosomes assembled *in vivo* on transcripts with variable number of introns, we examined a series of three related transcripts derived from the β-globin gene. Two of the genes contained increasing number of introns, while one gene encoded an exon only. The transcripts were each expressed in U2OS Tet-On stable cell clones that expressed the *β-globin* mini-genes that contain a series of MS2 sequence repeats in their 3′UTR (Brody et al., 2011). The three genes were under the transcriptional control of the inducible Tet-On system, and transcription was induced in the presence of the rtTA (Tet-On) transactivator expressed by the cells and the addition of doxycycline (dox) to the medium. The cell clones used were as follows: (i) E3, consisting of a *β-globin* mini-gene with three exons and two introns (Figure 1). Specifically, exon 3 was truncated and fused in-frame to a cyan fluorescent protein (CFP) coding region containing in its C-terminus the peroxisomal targeting tripeptide Ser-Lys-Leu (SKL). The mRNA therefore finally generated cytoplasmic cyan fluorescing peroxisomes. At the 3′-end of the gene, a series of 18 MS2 sequence repeats were added, thus providing high-affinity binding sites for the MS2 coat protein in the 3′UTR of the mRNA. (ii) E1, an intronless version of the mini-gene containing part of exon 3 only + CFP-SKL, together forming a single exon; and (iii) E6 with six exons and five introns, in which intron 2, flanked by the splice sites and part of exons 2 and 3, was multiplied (Figure 1). These cell clones were previously used in a study that followed transcription in living cells, showing that the transcriptionally active E3 and E6 genes recruited splicing factors and that the E3 and E6 mRNAs were co-transcriptionally spliced (Brody et al., 2011).

### E6 Transcripts Are Assembled in Supraspliceosomes

**FIGURE 2:**
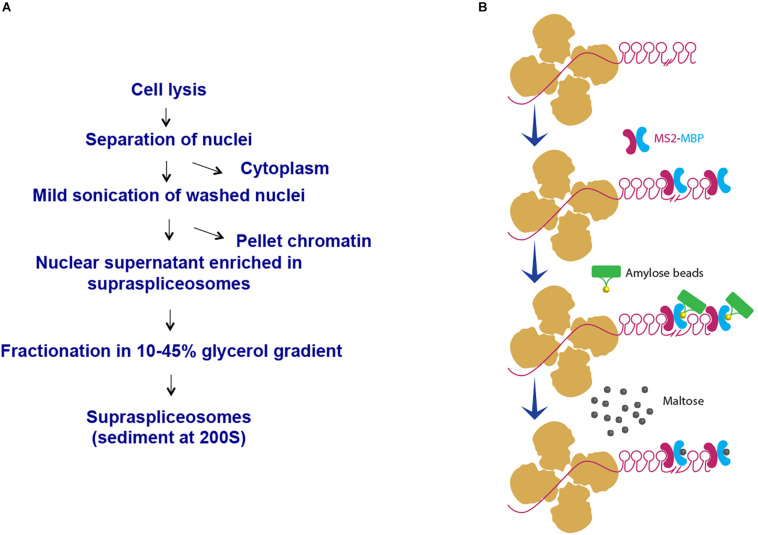
Scheme of affinity purification of MS2-tagged E6 supraspliceosomes. **(A)** Isolation of supraspliceosomes as previously described (Miriami et al., 1995; Azubel et al., 2006). Supraspliceosomes were isolated from U2OS cells stably expressing MS2-tagged E6 pre-mRNA by fractionation in a glycerol gradient of nuclear supernatants enriched with supraspliceosomes. **(B)** Affinity purification on amylose beads. Supraspliceosome fractions were pooled (fractions 8–10) and were incubated first with MS2–MBP. Next, the samples were incubated with amylose beads. After washing (×5), the bound E6 supraspliceosomes were eluted with maltose. As control, the procedure was repeated, except that buffer was added instead of MS2–MBP.

We have chosen to focus first on the E6-expressing cells as the E6 transcript has the highest number of introns relative to the E1- and E3-expressing cells. Also, preliminary analysis of the cells revealed that the E6-expressing cells had the highest level of expression of the β-globin MS2-tagged transcript (data not shown). We first used our protocol for isolation of endogenous spliceosomes under native conditions from cell nuclei (Figure 2A) to isolate complexes assembled in the E6 expressing U2OS cells. For this purpose, we prepared nuclear supernatants enriched with RNA Polymerase II transcripts, under physiological conditions and fractionated them in 10–45% glycerol gradients, as described previously (Miriami et al., 1995; Azubel et al., 2006). This method conserves higher-order splicing complexes, as formerly demonstrated by electron microscopy (Spann et al., 1989; Azubel et al., 2004; Azubel et al., 2006), which are associated with splicing factors (Miriami et al., 1995; Yitzhaki et al., 1996; Azubel et al., 2006; Markus et al., 2006; Heinrich et al., 2009; Sebbag-Sznajder et al., 2012; Yang et al., 2013; Kotzer-Nevo et al., 2014). To affinity purify complexes assembled on E6 transcripts, we followed the scheme detailed in Figure 2B. Recombinant MS2-MBP protein was added to the pooled supraspliceosome fractions (see section Materials and Methods), and the bound supraspliceosomes were further incubated overnight with amylose beads at 4°C. After washing, the bound supraspliceosomes were eluted by maltose. As a control, we repeated the same protocol with buffer only instead of MS2-MBP.

**FIGURE 3:**
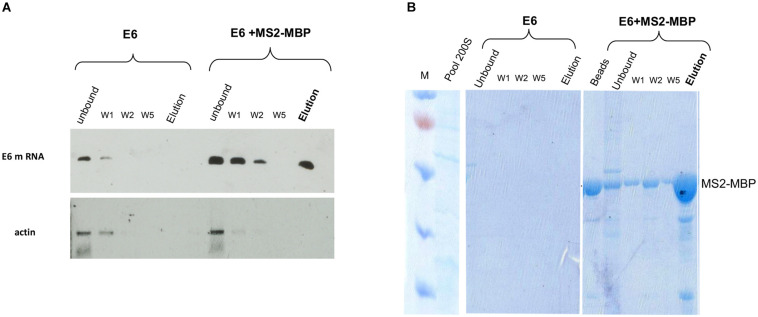
Specific affinity purification of MS2-tagged E6 supraspliceosomes. E6 spliceosomes were affinity-purified as described (Figure 2) and the eluted samples were analyzed by the following: **(A)** RT-PCR of β-globin mRNA of samples during the affinity purification procedure revealed specific binding and release (estimated yield 10%) of supraspliceosomes assembled on E6. As a control, buffer was added instead of the MS2-MBP to E6 supraspliceosomes. As an additional control, RT-PCR for actin mRNA showed that no mRNA was eluted. **(B)** Coomassie staining of 10% SDS-PAGE of samples isolated during the affinity purification procedure revealed the specificity of the affinity purification (“beads” designated fractions eluted with loading buffer). “M” is marker (PageRuler^TM^ Prestained Protein Ladder of Fermentas).

RT-PCR analysis of the affinity-purified supraspliceosomes revealed the association of E6 transcripts with supraspliceosomes (Figure 3A). The affinity purification is specific as E6 mRNA was specifically released by maltose from the amylose beads bound by MS2-MBP, while no eluted E6 mRNA was observed in the control experiment (Figure 3A). The binding to the amylose beads is specific to the MS2-tagged E6 mRNA, as RT-PCR analysis of endogenous actin mRNA showed no binding or elution. The specificity of the affinity purification was also confirmed by analysis of the eluted proteins by SDS PAGE followed by staining of the gel with Coomassie (Figure 3B), where eluted proteins were observed only in the samples bound by MS2-MBP and amylose. As expected, no MS2-MBP was observed in the control sample without it, yet no other proteins were observed in the eluted sample from the control experiment.

**FIGURE 4:**
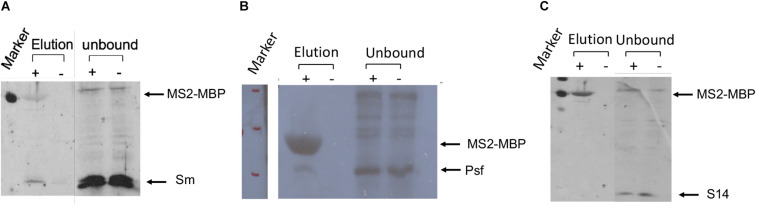
Splicing factors are associated with affinity-purified E6 supraspliceosomes. E6 supraspliceosomes were affinity-purified using MS2-MBP beads. **(A,B)** WB analysis using Y-12 anti-Sm Mabs and Anti-PSF Mab (SFPQ) (Thermo Scientific PageRuler Prestained Protein Ladder, #26616, marker was used; MW of MS2-MBP is 54.2 kDa) revealed that Sm proteins and PSF splicing factor are associated with the affinity-purified E6 supraspliceosomes, respectively, but they are not found in the control samples. **(C)** On the other hand, WB with Mabs against the ribosomal protein S14 revealed that S14 is not associated with the affinity-purified E6 supraspliceosomes.

The association of E6 with supraspliceosomes was further confirmed by WB analysis using anti-Sm Mabs (Y12), revealing that splicing components such as Sm proteins were associated with the affinity-purified E6 supraspliceosomes (Figure 4A). It should be noted that, as expected, the level of Sm proteins in the unbound sample was much higher than in the eluted sample. This is because the affinity purification is specific to the MS2-tagged supraspliceosomes, which constitute only a small fraction of the entire endogenous nuclear transcripts’ population, the majority lacking the MS2 tag. This was also exemplified in Figure 3A where endogenous actin supraspliceosomes were not affinity-purified. The association of E6 with supraspliceosomes was further confirmed by the association of E6 with the regulatory splicing factor PTB-associated splicing factor (PSF) (Figure 4B), also termed SFPQ (Shav-Tal and Zipori, 2002). On the other hand, WB analysis with antibodies directed against the ribosomal protein S14 (Figure 4C) revealed that S14 was not present in the affinity-purified E6 supraspliceosomes, further confirming the specificity of the affinity purification. These experiments revealed that E6 transcripts are assembled in supraspliceosomes.

### E1, E3, and E6 Transcripts Are Found in Supraspliceosomes

**FIGURE 5:**
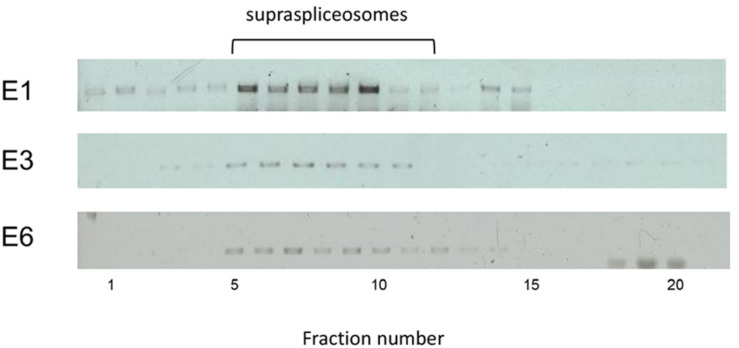
E1, E3, and E6 transcripts are assembled in supraspliceosomes. Nuclear supernatants enriched in supraspliceosomes were prepared from the stably transfected U2OS cells, expressing E1, E3, and E6 mRNA, respectively, and fractionated in a 10–45% glycerol gradient as previously described (Miriami et al., 1995; Azubel et al., 2006). 200S TMV particles that run in fractions 9 and 10 of analogous gradients were used for calibration. Supraspliceosomes peak in fractions 8–10. Aliquots from supraspliceosome fractions were analyzed by RT-PCR using primer pairs that flank exons 1–2 for E3 and E6, and primers of CFP-SKL for E1.

Each of the E1-, E3-, and E6-expressing cells was used to analyze the type of splicing complexes assembled on this series of transcripts containing an increasing number of introns and exons (E1, one exon and no intron; E3, three exons and two introns; E6, six exons and five introns). For this purpose, we used our protocol for isolation of endogenous spliceosomes under native conditions from cell nuclei and prepared nuclear supernatants enriched with RNA Pol II transcripts and fractionated them in 10–45% glycerol gradients (Figure 2A), as previously described (Miriami et al., 1995; Azubel et al., 2006). Next, we analyzed the distribution of each of the E1, E3, and E6 transcripts across its respective gradient by RT-PCR. Figure 5 reveals that despite the change in length and number of introns, E1, E3, and E6 transcripts peaked at the 200S region of the gradient, where supraspliceosomes (21 MDa) sedimented (Miriami et al., 1995; Müller et al., 1998; Azubel et al., 2006). The sedimentation patterns of E1, E3, and E6 were analogous to those of the phosphorylated SR proteins (Yitzhaki et al., 1996; Raitskin et al., 2002; Heinrich et al., 2009) and hnRNP G (Heinrich et al., 2009), which were shown previously to be predominantly associated with supraspliceosomes in these fractions. These results are consistent with our previous finding showing that diverse transcripts regardless of their length or number of introns are assembled in supraspliceosomes (Spann et al., 1989; Kotzer-Nevo et al., 2014). It also shows for the first time that even transcripts that are devoid of introns, like the E1 transcript, are assembled in supraspliceosomes, emphasizing their universal nature.

### Spliceostatin A (SSA) Inhibits Splicing but Not the Assembly Into Supraspliceosomes

To analyze how splicing inhibition affects the assembly of the supraspliceosome, we used Spliceostatin A (SSA). SSA is a methylated derivative of an anticancer bacterial metabolite FR901464. It inhibits splicing *in vitro* and *in vivo* by binding to SF3b, a component of U2 snRNP (Kaida et al., 2007). Previous studies showed that SSA inhibits spliceosome assembly *in vitro*, yet, all five spliceosomal U snRNPs and SSA were found associated with the inhibited spliceosomes (Roybal and Jurica, 2010). It was shown that SSA inhibits the binding of the SF3b 155-kDa protein to the pre-mRNA, resulting in reduced binding specificity of the U2 snRNP to the branch point, and causing some changes in alternative splicing (Corrionero et al., 2011). RNA-seq of transcripts after SSA treatment revealed that intron retention, namely, splicing inhibition, is the major effect of SSA on splicing (Carvalho et al., 2017; Yoshimoto et al., 2017). Furthermore, previous analysis of the effect of SSA on E6 transcripts in intact cells revealed that SSA treatment affects splicing, but not the rate of transcription. Yet, SSA obliterated the retention of E6 transcripts at the transcription site, resulting in rapid release of the transcript to the nucleoplasm (Brody et al., 2011), a release that can also be generated by the availability of splicing factors in the nucleoplasm (Hochberg-Laufer et al., 2019a). Treatment with SSA also results in partial pre-mRNA leakage (Kaida et al., 2007; Brody et al., 2011; Martins et al., 2011; Schmidt et al., 2011; Takemura et al., 2011; Carvalho et al., 2017; Yoshimoto et al., 2017).

**FIGURE 6:**
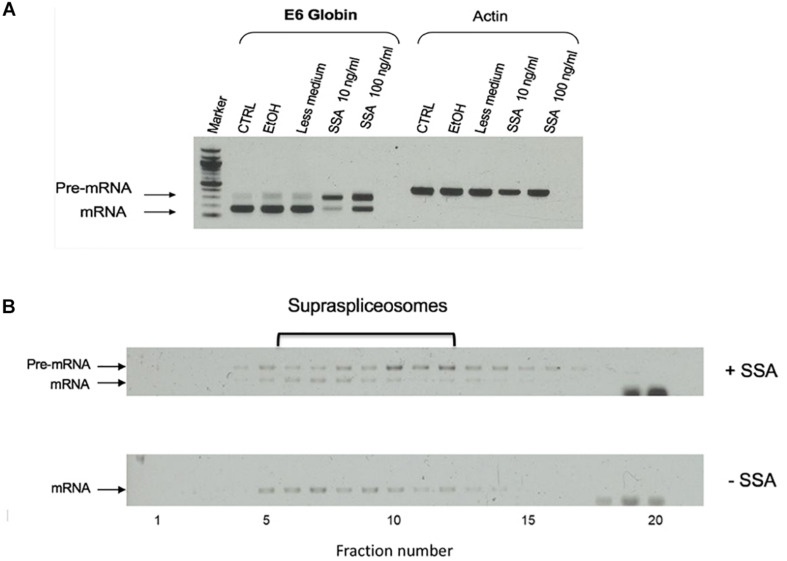
Effect of spliceostatin A (SSA) on E6 expression. **(A)** Effect of SSA on U2OS cells stably expressing the E6 transcript. Total RNA was extracted from E6 expressing U2OS cells treated with SSA (5 h), and the RNA was investigated by RT-PCR using primers from exons 1 and 2 for E6. Actin was used as a control. For comparison, RNA was extracted from untreated cells. Untreated cells incubated with ETOH was used as an additional control, as SSA was solubilized in ethanol. **(B)** Effect of SSA on E6 splicing in supraspliceosomes. Nuclear supernatants enriched in supraspliceosomes were prepared from U2OS E6 cells, treated or not with SSA (100 ng/mL, for 5 h), and were fractionated in a 10–45% glycerol gradient and gathered (bottom to top) in 20 fractions. 200S TMV particles that run in fractions 9 and 10 of analogous gradients were used for calibration. Aliquots from each gradient fraction were examined by RT-PCR with primer pairs that flank exons 1–2 of E6.

To test the effect of SSA on the expression of E6 transcripts, we first incubated the cells for 5 h with SSA at 10 or 100 ng/mL and prepared total RNA from the treated cells. As controls, we used untreated cells and cells incubated with ethanol, since SSA is dissolved in ethanol. Figure 6A shows that while untreated cells expressed mainly mature E6 mRNA, after treatment with SSA at 10 ng/mL, the E6 mRNA was predominantly unspliced. Treatment with 100 ng/mL SSA decreased the percentage of E6 pre-mRNA. This effect of increasing amount of SSA on splicing is not clear. It is possible that additional effects of SSA on gene expression play a role here, such as the coupling of transcription and splicing (e.g., it has been shown that treatment with SSA at 100 ng/mL decreases the phosphorylation of Ser2 in Pol II CTD, causes early dissociation of Pol II, and decreases phospho-Ser2 level of chromatin-bound Pol II; Koga et al., 2015). Thus, it is possible that high SSA levels affect transcription in addition to splicing, yet other explanations cannot be ruled out at this stage. Next, we tested the effect of SSA on the E6 supraspliceosomes. Supraspliceosomes were prepared from the E6-expressing U2OS cells, either treated or untreated with 100 ng/mL SSA for 5 h, and fractionated in glycerol gradients. Figure 6B shows that E6 supraspliceosomes from SSA-treated and untreated cells were found in supraspliceosomes that sediment at 200S in the glycerol gradients. While E6 supraspliceosomes from untreated cells assembled E6 mRNA, E6 supraspliceosomes from SSA-treated cells portrayed mainly E6 pre-mRNA and a lower percentage of E6 mRNA.

**FIGURE 7:**
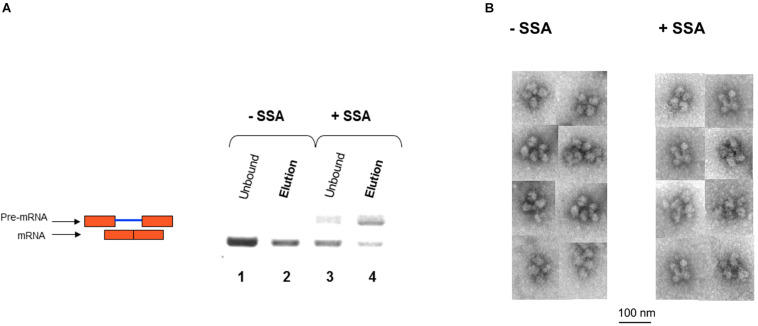
SSA inhibits splicing but not supraspliceosome assembly. **(A)** Affinity-purified supraspliceosomes from SSA-treated cells are enriched with E6 pre-mRNA. Supraspliceosomes isolated from E6 expressing U2OS cells treated with SSA were affinity-purified as described above, using the MS2-MBP protein, binding to amylose beads and specific elution of E6 supraspliceosomes with maltose. RNA was extracted from E6 supraspliceosomes incubated or not with SSA (100 ng/mL, for 5 h), and from the unbound material, and analyzed by RT-PCR using primer pairs that flank exons 1–2 of E6. E6 pre-mRNA and mRNA are schematically drawn on the left. **(B)** SSA did not affect the assembly of the E6 transcript into supraspliceosomes. EM visualization of supraspliceosomes from E6 U2OS cells treated or not with SSA. A gallery of negatively stained supraspliceosomes observed in aliquots from the supraspliceosome peak fractions of E6 U2OS cells treated or not with SSA.

We next affinity-purified E6 supraspliceosomes from SSA-treated and untreated cells (Figure 7A). Supraspliceosomes were found assembled on mature E6 mRNA in untreated cells. After treatment with SSA, affinity-purified E6 supraspliceosomes were mainly assembled on E6 pre-mRNA, while a small percentage was assembled on E6 mRNA. These studies revealed that SSA inhibits splicing, but does not interfere with supraspliceosome assembly. This finding is further confirmed by electron microscopy visualization of aliquots from the 200S peak region of the glycerol gradients where supraspliceosomes sediment. Supraspliceosomes, composed of four native spliceosomes, were visualized in both treated and untreated cells, and no significant structural changes could be visualized in the SSA-treated supraspliceosomes (Figure 7B).

**FIGURE 8:**
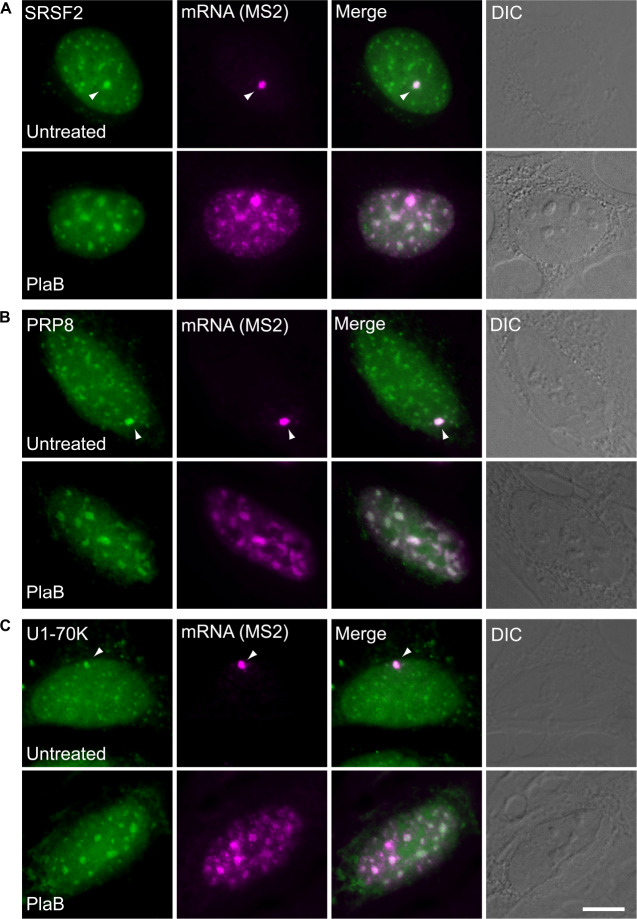
Splicing factors continue to be recruited to the E6 mRNA under splicing inhibition conditions. The recruitment of several RNA processing factors to the actively transcribing E6 gene was examined in cells stably expressing BACs that transcribe GFP fusions of either **(A)** SRSF2, or **(B)** Prp8, or **(C)** U1-70K (green) under normal and splicing inhibition by Pladienolide B (PlaB). The active site of transcription of the E6 gene was detected by RNA FISH with a Cy5-labeled probe that hybridizes to the MS2 region of the E6 mRNA (magenta). Arrowheads point to the actively transcribing genes. DIC is in gray. Bar = 5 μm.

**FIGURE 9:**
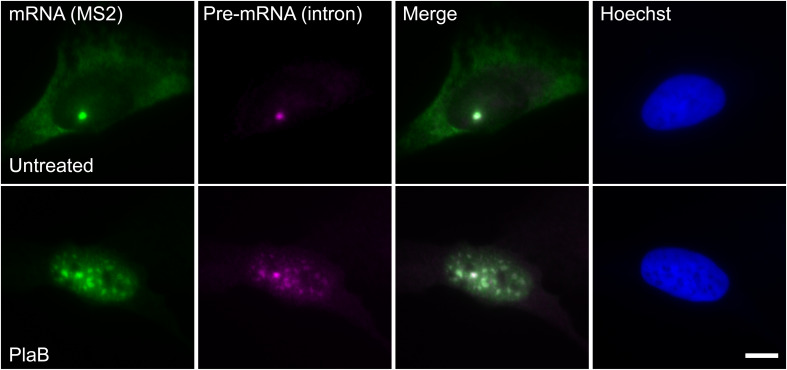
E6 mRNA accumulated in the nucleus under splicing inhibition. The distribution of E6 mRNA under normal (top) and splicing inhibition conditions by Pladienolide B (PlaB) (bottom) was detected by RNA FISH using a Cy5-labeled probe that hybridizes with the MS2 region of the E6 mRNA (green), and a Cy3-labeled probe that binds to the intron of the E6 mini-gene (magenta). Hoechst DNA counterstain is in blue. Bar = 10 μm.

These findings were next corroborated in intact cells. We examined whether splicing factors can indeed continue to co-transcriptionally assemble on the E6 mRNAs during transcription under normal and splicing inhibition conditions. We used U2OS Tet-On stable cell lines that contain a stable integration of the E6 gene in one gene locus. The gene integration forms a tandem array of the gene and, upon activation with dox, a single spot of the active E6 gene can be detected by RNA FISH with a probe to the MS2 repeats in the 3′UTR of the transcript (Brody et al., 2011). This is the active site of transcription on which we wanted to detect whether co-transcriptional recruitment of splicing factors occurs, as previously described (Huranova et al., 2010; Brody et al., 2011; Hochberg-Laufer et al., 2019a,b). In order to detect the splicing factors in intact cells, we used E6 cells with additional stable integrations of bacterial artificial chromosomes (BACs) (Figures 8A–C) containing the full gene body of either the SR protein SRSF2 (SC35), or two snRNP components, U1-70K (part of the U1 snRNP, which binds to the 5′-splice site) and PRP8 (part of U5 snRNP, which is part of the U4/U6.U5 triple-snRNP) tagged with GFP in the C-terminal (Poser et al., 2008). Using RNA FISH that detects the MS2-tagged E6 mRNA together with the staining of the cells with GFP-tagged SRSF2, or U1-70K or Prp8, we found that these splicing factors were recruited to the transcriptionally active E6 gene under normal conditions, as expected. When splicing was inhibited using Pladienolide B, which inhibits splicing by interaction with the SF3B complex [similar to SSA, (Kotake et al., 2007)], the E6 pre-mRNAs accumulated in the nucleus (Figure 9), specifically in nuclear speckles that are known to contain splicing factors, as is known to occur for unspliced transcripts. Importantly, the splicing factors continued to be recruited to the transcribing genes and so continued to assemble on the pre-mRNAs under splicing inhibition conditions, in agreement with the biochemical data showing that supraspliceosomes assembled on these mRNAs under all conditions.

## Discussion

Although most of RNA Pol II transcribed pre-mRNAs are multi-intronic, the process of spliceosome assembly on such pre-mRNAs is at present not well understood. To address this question, we chose here to examine spliceosome assembly *in vitro* and *in vivo* on a series of three related transcripts: two of the transcripts contained increasing number of introns derived from the β-globin gene (E6 with six exons and five introns, E3 with three exons and two introns) and one that had only one exon and no introns. Each transcript had multiple MS2 sequence repeats that can be bound by the MS2 coat protein and therefore be used for both affinity purification and visualization in intact cells. We showed that E6 transcripts are assembled in supraspliceosomes composed of four native spliceosomes joined together by the transcript. This was confirmed by analyzing isolated and affinity-purified complexes and by studies in intact cells showing the association of E6 with splicing factors. We further demonstrated that this series of transcripts, namely, E6 with five introns, E3 with two introns, and E1 with no intron, are assembled in supraspliceosomes. These findings corroborated our previous findings showing that Pol II transcripts are assembled into 21-MDa supraspliceosomes, regardless of their number of introns or length (reviewed in Sperling et al., 2008; Shefer et al., 2014; Sperling, 2017). For example, SMN transcripts, having eight introns, were shown sedimenting at 200S with supraspliceosomes, and were demonstrated as assembled in supraspliceosomes by immunoprecipitation using anti-Sm antibodies, further showing that both splicing isoforms, with and without exon 7, were assembled in supraspliceosomes (Sebbag-Sznajder et al., 2012). An additional example is the case of PP7-tagged AdML transcripts that contain one intron. Analysis of the affinity-purified PP7 tagged splicing complexes assembled on this AdML transcript *in vivo* demonstrated that they are assembled in supraspliceosomes (Kotzer-Nevo et al., 2014). The supraspliceosome, composed of four native spliceosomes, can splice four introns at one setting. Because all pre-mRNAs are found assembled in supraspliceosomes, independent of their number of introns, we suggest that splicing of a pre-mRNA having more than four introns likely occurs through the movement of the pre-mRNA through the supraspliceosome in a “rolling model” manner. In the case of pre-mRNA with one intron, or with less than four introns, which are also assembled in supraspliceosomes (Azubel et al., 2006; Kotzer-Nevo et al., 2014), or with intronless transcripts, as shown here, it is likely that the interactions of the transcript with the native spliceosomes are appropriate to keep the supraspliceosome structure. Supraspliceosomes harbor all five spliceosomal U snRNPs (Miriami et al., 1995; Azubel et al., 2006; Kotzer-Nevo et al., 2014) and splicing factors (Yitzhaki et al., 1996; Markus et al., 2006; Chen et al., 2007; Heinrich et al., 2009; Yang et al., 2013; Kotzer-Nevo et al., 2014). The finding of regulatory splicing factors within supraspliceosomes is in line with their function in splicing regulation and alternative splicing (Heinrich et al., 2009; Sebbag-Sznajder et al., 2012). Supraspliceosomes also contain all the additional factors required for pre-mRNA processing, including 5′-cap components, 3′-end processing components, and A-to-I RNA processing, in addition to splicing and alternative splicing components (Raitskin et al., 2001; Raitskin et al., 2002), portraying them as the nuclear pre-mRNA processing machine. This likely explains the finding that a transcript with no introns (E1) is also assembled in supraspliceosomes. Here, we not only confirmed our previous findings but also show that a transcript lacking an intron is also assembled in supraspliceosomes. This result is in agreement with our previous finding that a PP7-tagged AdML transcript assembled on mature AdML is assembled in supraspliceosomes (Kotzer-Nevo et al., 2014).

We next examined how splicing inhibition affects the assembly into supraspliceosomes. For this aim, we used spliceostatin A (SSA), which binds to the SF3b part of U2 snRNP and inhibits splicing *in vitro* and *in vivo* (Kaida et al., 2007; Lo et al., 2007). Treatment with SSA reduces the binding specificity of the U2 snRNP to the branch point, resulting in some changes in alternative splicing (Corrionero et al., 2011). Focusing on E6 transcripts, we show that treatment with SSA inhibits splicing and increases the percentage of E6 pre-mRNA. However, this intron retention did not affect the assembly of E6 pre-mRNA into supraspliceosomes composed of four native spliceosomes connected by the transcript. This was confirmed for isolated E6 supraspliceosomes, using ultracentrifugation, affinity purification, and electron microscopy. These results were further corroborated by studies in intact cells, using Pladienolide B, which inhibits splicing by interaction with the SF3B complex (similar to SSA; Kotake et al., 2007), showing that the association of E6 pre-mRNA at the active gene locus with essential splicing factors was not affected by the inhibition of splicing, yet it resulted in nuclear accumulation of the E6 spliceosomes. These latter findings are consistent with previous observations of nuclear accumulation of pre-mRNA resulting from treatment with SSA probably due to lack of appropriate export signals that are assembled during regular splicing (Carvalho et al., 2017; Yoshimoto et al., 2017).

The splicing complex is dynamic, undergoing chemical changes during the two steps of the splicing reaction, involving dynamic changes in U snRNA:U snRNA, U snRNA:pre-mRNA, and protein:RNA interactions, accompanied by local structural changes as revealed by the recent high-resolution structures of spliceosome intermediates (reviewed in Will and Luhrmann, 2011; Papasaikas and Valcarcel, 2016; Fica et al., 2017; Shi, 2017a,b; Wilkinson et al., 2018; Plaschka et al., 2019; Yan et al., 2019). In agreement with that, the supraspliceosome is a dynamic complex, as splicing and alternative splicing occur in supraspliceosomes (Azubel et al., 2006; Sebbag-Sznajder et al., 2012). We have shown here that both pre-mRNA and spliced transcripts are assembled in supraspliceosomes, as demonstrated for E6 pre-mRNA and mRNA that are assembled in supraspliceosomes composed of four native spliceosomes joined together by the transcript. This finding and the results showing that a series of three related transcripts, two having a growing number of introns derived from the β-globin gene (two and five introns, respectively) and a third having only one exon and lacking an intron, are assembled in supraspliceosomes confirm the generality of the supraspliceosome.

In previous studies, we have shown that the supraspliceosome is assembled on one transcript (Sebbag-Sznajder et al., 2012). We have further demonstrated that the pre-mRNA is linking the four native spliceosomes of the supraspliceosome, as specific cleavage of the pre-mRNA using RNase H yielded native spliceosomes (Azubel et al., 2004). In this study, we further show that the transcript whether spliced or not is connecting the four spliceosomes of the supraspliceosome. Namely, an mRNA can also connect the four native spliceosomes, as in the case of E1 transcripts, which lack an intron, and yet they are also found assembled in supraspliceosomes. This finding is supported by previous studies showing that affinity-purified transcripts of AdML mini-gene having either one intron or spliced were found assembled in supraspliceosomes (Kotzer-Nevo et al., 2014).

It should be noted that our previous studies have shown that both the native spliceosome and the supraspliceosome contained all five spliceosomal U snRNPs (Azubel et al., 2006) and that the supraspliceosome harbors the five spliceosomal U snRNPs throughout all stages of the splicing reaction (Kotzer-Nevo et al., 2014). This is in contrast to changes in composition during spliceosome assembly observed *in vitro* (Wahl et al., 2009; Will and Luhrmann, 2011). It is possible that supraspliceosomes and native spliceosomes harbor additional components to those of intermediate complexes assembled *in vitro*, which help keep them together. The remodeling of the spliceosome during the splicing reaction is regulated by a vast dynamic network of RNA:RNA, protein:protein, and RNA:protein interactions. These alterations might not require extensive variations in the general shape of the splicing complex, but might be accommodated by limited conformational variations. It should be pointed out that the detailed dynamic changes that happen along the two steps of the splicing reaction cannot be visualized at this resolution of EM visualization of supraspliceosomes, and higher-resolution studies are required for that.

## Data Availability Statement

The raw data supporting the conclusions of this article will be made available by the authors, without undue reservation, to any qualified researcher.

## Author Contributions

RS, JS, and YS-T developed the project and designed the experiments. NS-S performed the supraspliceosome experiments. YB generated the cell clones. HH-L performed the imaging experiments. RS and YS-T wrote the manuscript.

## Conflict of Interest

The authors declare that the research was conducted in the absence of any commercial or financial relationships that could be construed as a potential conflict of interest.
